# Advances in Non-Coding RNA Sequencing

**DOI:** 10.3390/ncrna7040070

**Published:** 2021-10-30

**Authors:** Julia Micheel, Aram Safrastyan, Damian Wollny

**Affiliations:** RNA Bioinformatics/High Throughput Analysis, Faculty of Mathematics and Computer Science, Friedrich Schiller University, 07743 Jena, Germany; julia.micheel@uni-jena.de (J.M.); aram.safrastyan@uni-jena.de (A.S.)

**Keywords:** non-coding RNA, next-generation sequencing, transcriptomics, diagnostics, liquid biopsy

## Abstract

Non-coding RNAs (ncRNAs) comprise a set of abundant and functionally diverse RNA molecules. Since the discovery of the first ncRNA in the 1960s, ncRNAs have been shown to be involved in nearly all steps of the central dogma of molecular biology. In recent years, the pace of discovery of novel ncRNAs and their cellular roles has been greatly accelerated by high-throughput sequencing. Advances in sequencing technology, library preparation protocols as well as computational biology helped to greatly expand our knowledge of which ncRNAs exist throughout the kingdoms of life. Moreover, RNA sequencing revealed crucial roles of many ncRNAs in human health and disease. In this review, we discuss the most recent methodological advancements in the rapidly evolving field of high-throughput sequencing and how it has greatly expanded our understanding of ncRNA biology across a large number of different organisms.

## 1. The Dawn of Molecular Biology and Nucleic Acid Sequencing

The central dogma of molecular biology that Francis Crick proposed in 1957 described a model for the flow of information between genes and proteins [1]. In subsequent years, much effort has been undertaken to elucidate the composition of RNA subtypes and their involvement in information flow towards protein synthesis and beyond. Given its central role in protein synthesis, the protein-coding messenger RNA (mRNA) has been extensively studied since its discovery in 1960 [2]. Additionally, in the 1950s and 1960s, non-coding RNAs (ncRNAs) such as transfer RNA (tRNA) [3], ribosomal (rRNA) [4], and small nuclear RNA (snRNA) [5], were discovered and their cellular roles elucidated. Meanwhile, the remaining parts of the genome were for a long time considered to be “evolutionary junk” [6,7], or “selfishly” propagating throughout the genome without contribution to the phenotype [8]. The discovery of the first regulatory ncRNAs such micF in *Escherichia coli* in 1984 [9], as well as the first eukaryotic long ncRNAs H19 [10], and Xist [11], and microRNA (miRNA) lin-4 in *Caenorhabditis elegans* [12], in the early 1990s slowly revealed a more complex role of ncRNAs in cells.

In parallel to the discoveries of diverse RNAs and their roles in the field of molecular biology, a novel technology for the determination of the sequences of nucleic acids appeared on the horizon. The first DNA sequencing approach has been developed by using a chain termination approach established by Sanger and colleagues in 1977 [13]. Based on this method, the first viral genome could be sequenced as early as 1977 [14], and bacterial genome sequences could also be determined in this way [15]. However, the effort required for complex eukaryotic genomes was much greater. Sequencing a large genome such as the human genome with the techniques at hand at that time required a coordinated effort by a large consortium. The so-called Human Genome Project involved twenty universities and research centers from six countries and took 13 years to complete [16,17]. In an effort to increase the throughput and thus the number of bases sequenced in a given time interval, next generation sequencing methods were developed. In 2005, the first next generation sequencing (NGS) technique named pyro sequencing was introduced, which enabled massively parallel sequence analysis [18]. Beyond the characterization of single cell types or cell lines, efforts by large groups of scientists such as the Encyclopedia Of DNA Elements (ENCODE) project [19] or the Functional Annotation of the Mouse/Mammalian Genome (FANTOM) consortium [20,21], enabled comprehensive insights into the information content of the mammalian genome and transcriptome.

**Figure 1:**
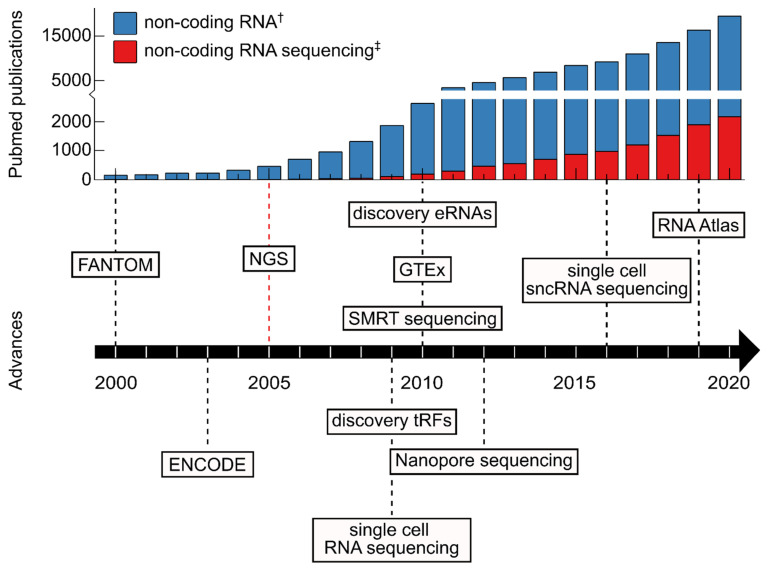
Important advances in non-coding RNA research over the last two decades. The bar chart presents the numbers of publications with the topics “non-coding RNA” and “non-coding RNA sequencing” per year according to the timeline represented as an arrow. (^†^ Pubmed search for “ncRNA” OR “miRNA” OR “piRNA” OR “snoRNA” OR “snRNA” OR “lncRNA” OR “circRNA” OR “non-coding RNA” OR “micro RNA” OR “piwi-interacting RNA” OR “small nucleolar RNA” OR “small nuclear RNA” OR “long non-coding RNA” OR “circular RNA” of 22/10/2021; ^‡^ Pubmed search for “ncRNA” OR “miRNA” OR “piRNA” OR “snoRNA” OR “snRNA” OR “lncRNA” OR “circRNA” OR “non-coding RNA” OR “micro RNA” OR “piwi-interacting RNA” OR “small nucleolar RNA” OR “small nuclear RNA” OR “long non-coding RNA” OR “circular RNA” AND “sequencing” of 22 October 2021). Selected milestones in non-coding RNA research are displayed on the timeline arrow. The dates for consortia (FANTOM [20], ENCODE [19], GTEx (GTEx Consortium 2013) [25]) were based on the funding year. Advances in sequencing techniques were dated based on their commercial availability (next generation sequencing (NGS) starting with 454 sequencing [18], SMRT sequencing [26], Nanopore sequencing [27]). Novel library preparation methods (single cell RNA sequencing [28], single cell sequencing of small non-coding RNA (sncRNA) [29], databases (RNA Atlas [30]), and newly discovered ncRNA species (tRNA-derived fragments (tRFs) [31], eRNAs [32]) were dated based on their publication years.

The latest major advance in sequencing technology is the development of long-read sequencing (also known as “third generation” sequencing). Long-read sequencing is largely brought about by two novel technologies: Single-molecule real-time (SMRT) sequencing and nanopore sequencing, which are commercialized by Pacific Biosciences and Oxford Nanopore respectively (Figure 1). SMRT sequencing is an optical method that utilizes a zero-mode waveguide in order to observe single nucleotides of DNA while being incorporated by a DNA polymerase [22]. In contrast, nanopore sequencing is based on measuring changes of electrical current while nucleic acids are passing through a protein channel [23]. One major advantage that these highly different technologies have in common is the ability to produce reads that are substantially longer than usually obtained through NGS sequencing approaches [24]. This is significant, because long reads substantially improve de novo assembly of sequenced genomes, aid the identification of transcript isoform as well as structural variants.

**Figure 2:**
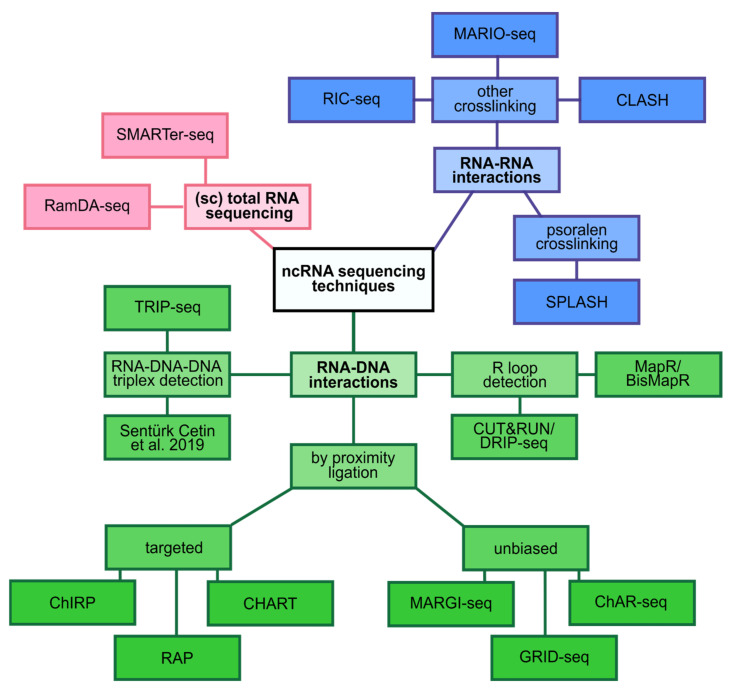
Overview of recent ncRNA sequencing techniques. The scheme summarizes techniques for single-cell total RNA sequencing (RamDA-seq [84], SMARTer-seq [86]), sequencing of RNAs interacting with DNA by proximity ligation (ChIRP [87], RAP [88], CHART [89], MARGI-seq [90], GRID-seq [91], ChAR-seq [92]), interacting in triplexes (TRIP-seq [93], Sentürk Cetin et al. [94]) and R loops (CUT&RUN [95], DRIP-seq [96], MapR [97], BisMapR [98]) as well as interacting with other RNAs (RIC-seq [99], MARIO-seq [100], CLASH [101], SPLASH [102]).

The development of different sequencing techniques enables faster and more cost-efficient sequencing and improved quality of sequencing data [33]. Additionally, advancing protocols for library preparation allowed for the detection of quantification of molecules present at minuscule amounts enabling the exhaustive characterization of e.g., genomic mutation landscape or the RNA molecules present in the transcriptome of cell types of interest. The enormous increase in information gain as a consequence of sequencing advances naturally has also advanced the field of ncRNAs (Figure 1 and Figure 2).

This review summarizes the recent development in ncRNA sequencing and describes the impact sequencing technology has had on the field of ncRNA biology. We focus on how RNA sequencing has been an important tool to uncover novel ncRNAs (Table 1) as well as their functional roles in human health and disease. Further, we emphasize that sequencing technology has greatly expanded our understanding of ncRNA biology across the phylogenetic tree, far beyond mammalian organisms.

## 2. Advancing Our Understanding of ncRNA Biology through RNA Sequencing

While genome sequencing efforts came to fruition upon the turn of the century, one major question which remained open was: How much of the genome is transcribed? In order to address this question, the RIKEN institute decided to start a consortium called FANTOM consortium [74]. The goal of this international collaborative effort was to systematically determine the full coding potential of the mouse genome. These efforts resulted in the functional annotation of a full-length mouse cDNA collection identifying novel genes and detecting novel alternative splice isoforms [75]. In subsequent years, the FANTOM consortium also developed invaluable tools for ncRNA research. One example was the effort to determine transcriptional start sites across the genome. Promoters can be detected by sequencing the 5′ends of full-length cDNA libraries and subsequently map these to the genome. In order to get high quality sequences from the 5′ends of transcripts in a high throughput fashion a technique called cap analysis gene expression (CAGE) was developed [76,77]. Using this method, it became clear that a lot more RNAs are present in the mammalian transcriptome than previously anticipated [74]. This notion was independently confirmed, arguing that three quarters of the human genome is capable of being transcribed [78]. It was, however, extrapolated that most ncRNA are present at less than one copy per cell, which provoked the question whether most ncRNAs might be transcriptional “junk” [79]. Another explanation would be that only a subset of the investigated cells expresses certain ncRNAs [74], which could not be deciphered by bulk RNA sequencing.

### 2.1. Sequencing Total RNA from Single Cells

In general, progress in understanding biology through RNA sequencing rests heavily on the shoulders of technological advancements. An enduring goal of the field is to increase the detection sensitivity in order to gain information when only small quantities of RNA or DNA are available. Within the last decades, substantial progress has been made on that front, making it possible to sequence the RNA content even from single cells. The field of single cell transcriptomics has subsequently greatly enhanced our understanding of cellular heterogeneity across many tissues [80]. This led to numerous discoveries such as previously neglected cell types [81], or altered phenotypes in subsets of neurons as a consequence of disease [82]. With time, the field broadened significantly in terms of which organs or species were studied [83]. However, in contrast to the increasingly exotic organs and organisms that this technology was applied to, the field was rather conservative in terms of which molecules were profiled. Given that the most used protocols target only polyadenylated RNAs, the vast majority of studies sequenced mostly mRNAs of cells. Thus, with the exception of certain polyadenylated ncRNAs such as some long non-coding RNAs (lncRNAs), the ncRNA landscape was mostly neglected. One technical explanation for the neglect could be the fact that many ncRNAs are present at only low copy numbers and are thus challenging to detect [79].

This started to change with the first demonstration of single-cell sequencing of the small RNAs [29]. With this technology at hand, the authors profiled human embryonic stem cells (hESCs) and demonstrated that the expression of certain miRNAs was highly heterogeneous in primed hESCs but not in native hESCs [29]. Although this study only looked at a limited number of ncRNA species, it was able to demonstrate that previously neglected ncRNAs are indeed contributors to the molecular heterogeneity of cells. In an attempt to investigate how other ncRNA species contribute to cellular heterogeneity, another method called random displacement amplification sequencing (RamDA-seq) was developed [84], (Figure 2). By using a combination of semi-random hexamers and oligo-dT primers, the authors were able to extract an impressive amount of information and presented the first full-length total RNA-seq approach from single cells. Notably, RamDA-seq detected hundreds of dynamically regulated ncRNAs including enhancer RNAs (eRNAs) and their dynamic regulation during cellular differentiation. One potential downside of the RamDA-seq protocol is that it does not deplete rRNAs. A total RNA seq approach in which the rRNA molecules are kept will spend a lot of sequencing reads on rRNA molecules, as they are the most abundant RNA biotype in all cells [85]. In order to overcome this issue, a total RNA-seq protocol called SMARTer designed specific ribosomal cDNA removal probes [86]. Using this protocol, the authors were able to more efficiently detect ncRNAs such as circular RNAs (circRNAs) without specific enrichment for them by e.g., degradation of linear RNAs.

Beyond simply probing which ncRNA molecules are present in any given sample, sequencing technology can also give insights into the functional role of ncRNAs. On the computational side, tools are being developed to help us to understand what the myriad of ncRNA sequences obtained by sequencing reveals about ncRNA function. Recent computational tools based on deep learning were developed to improve sequence classification into ncRNA classes or infer function of short ncRNAs transcripts based on the sequences alone [103,104].

One of the most studied roles for ncRNAs is the regulation of the transcriptome. This can take place at the different stages such as the transcriptional, post-transcriptional or chromatin level [105]. Since these processes are mediated by physical interaction of ncRNAs with either DNA, RNA or protein, methods for detecting these interactions are therefore very insightful. Although the role of ncRNAs can obviously also be studied using traditional approaches, sequencing technology represents a powerful asset for the comprehensive analysis of the global cellular landscape.

### 2.2. Transcriptional Regulation

ncRNAs have the ability to directly interact with the transcriptional machinery and thereby regulate gene expression. One of the most comprehensively studied examples of that is the mechanism of sex-chromosome dosage compensation [106]. The inactivation of one of the X chromosomes in female mammals is triggered by the ncRNA XIST, which coats and silences the Xi chromosome in a PRC2 dependent manner [107,108]. The importance of XIST for this process was affirmed by the demonstration that X inactivation is dependent on XIST [109]. Yet, despite its central role in this process, the mechanism by which XIST spreads across the X chromosome was only recognized with the help of sequencing technology [88]. In an elegant study, DNA regions that were associated with XIST RNA molecules in vivo were purified and sequenced. Time series experiments demonstrated that XIST spreads rapidly to distal regions in the genome. These regions are, however, in close physical proximity due to the three-dimensional structure of the chromosome [88]. This finding demonstrates the power of applying sequencing technology to mechanistic questions of ncRNA function.

In contrast to transcriptional silencing, sequencing ncRNAs also led to important insights into transcriptional activation. One example for this is the discovery of eRNAs. Enhancers are genomic regions that activate transcription from a distance and are often marked by histone modifications such as H3K27Ac [110]. Interestingly, a study that aimed to sequence the DNA regions that are bound by the transcriptional activator RNAPII, found that RNAPII binds a lot more frequently to enhancer regions than previously recognized [32]. Since this finding strongly indicated that RNA transcription may occur at enhancers, the authors performed RNA sequencing and indeed found RNA molecules derived from enhancer regions [32]. Moreover, eRNA expression levels correlated with mRNA transcription from nearby genes, suggesting a role of eRNAs in transcriptional activation.

### 2.3. Regulation on the Chromatin Level

A major determinant of transcriptional regulation is the chromatin state [111]. Hence, methods to determine the global interplay between ncRNA, DNA and protein components of chromatin were sought after. Recently, a number of technical advancements opened the door towards a comprehensive understanding of the involvement of ncRNA in chromatin mediated gene regulation.

One of the first methods that aimed to map the global RNA-chromatin interactome in an unbiased fashion was named global RNA interactions with DNA by deep sequencing (GRID-seq) [91]. GRID-seq uses a bivalent linker to proximity ligate RNA to DNA in situ followed by bead capture and library preparation for high-throughput sequencing. This strategy uncovered defined groups of interactions between RNA and chromatin regions that are specific to the distinct gene expression profiles of different cell types [91]. Two similar approaches called chromatin-associated RNA sequencing (ChAR-seq) and Mapping RNA-genome interactions (MARGI), all of which are based on proximity ligation, complement GRID-seq to reveal chromatin associated RNAs [90,92]. Interestingly, GRID-seq was able to distinguish between cis-acting and trans-acting groups of RNAs. Furthermore, the exclusive detection of XIST on the X chromosome [88], and the enriched detection of spliceosome RNAs in regions of active transcription [92], further demonstrate the great potential of these approaches. It should be noted, however, that these methods might be biased to some extent since highly abundant RNAs will be detected at higher rates compared to lower expressed RNAs.

Another set of methods that have been termed “hybridization capture methods” detect interaction sites of ncRNA with chromatin in a genome-wide fashion [112]. Methods such as capture hybridization analysis of RNA targets (CHART) [89], chromatin isolation by RNA purification (ChIRP) [87], and RNA affinity purification (RAP) [88], are all based on the hybridization of biotin-labelled antisense oligos to target (nc)RNAs. The tagged antisense oligos can then be captured using streptavidin beads and the eluted molecules can subsequently be determined by high-throughput sequencing (RNA, DNA) or by mass spectrometry (protein) [112].

Besides direct interaction with the proteins that bind to DNA, ncRNAs have also been shown to directly interact with DNA and thereby affect chromatin accessibility. One example of these direct interactions are triple helices called RNA–DNA–DNA triplexes [113]. Recently, a method called triple helix-sequencing (TRIP-seq) was developed to investigate triplex-forming sequences in cells and was able to reveal triplex binding sites with respect to nucleosome positions in vivo [93]. Similarly, Sentürk Cetin and colleagues developed a fast and cost-effective method to isolate, sequence and characterize DNA-RNA triplexes [94]. Sequencing technologies like these in combination with powerful computational tools to predict the triplex-forming potential of RNAs within particular target DNA regions [114], will greatly advance this research field, which is only beginning to uncover the intricate mechanisms of triplex mediated gene expression regulation.

Another form of RNA-DNA hybrid, which can involve mRNA as well as ncRNAs, resulting in the displacement of single-stranded DNA are so called R-loops. Mounting evidence accumulated in recent years suggests a number of regulatory functions of R-loops in gene expression [115]. Initially, R-loops were believed to result as a detrimental bi-product of transcription. However, first genome-wide profiling attempts of R-loops demonstrated that they were non-randomly distributed across the genome with particular enrichment in retrotransposons, telomeres or genomic regions coding for ribosomal RNA [96,116]. Continuous efforts attempt at improving genome wide R-loop profiling. The most recent development on that front called BisMapR represents an antibody-independent approach that utilizes bisulfite chemistry to produce strand-specific genome-wide profiles and thereby, achieves greater resolution and is faster than previous methods [98]. Another method for R-loop detection by sequencing that does not rely on bisulfite conversion is called MapR [97]. MapR utilizes the sensitivity and speed of a technique called Cleavage Under Targets and Release Using Nuclease (CUT&RUN) [95] and combines it with the specificity of RNAse H for the detection of DNA:RNA hybrids. The sensitivity of MapR enables the obtainment of genome-wide coverage from very little input material in a short amount of time.

### 2.4. Post-Transcriptional Regulation

One of the most extensively studied ways to regulate the transcriptome post transcription is through miRNAs [117]. According to current estimates, 2300 mature miRNAs are encoded in the human genome [118]. They consist of ~22 nt [119], and work as negative regulators of transcripts via degradation initiation or translation inhibition (dependent on extent of complementary base pairing) [120]. Since the discovery of the first miRNA in nematodes, a large number of miRNAs in different species have been discovered as evident by databases such as miRbase (Table 1). The characterization of miRNAs and the small RNA transcriptome in general was revolutionized by the myriad of small RNA sequencing technology developments in the last years that have been extensively reviewed elsewhere [121].

Beyond the mere description of which miRNAs are expressed under any given circumstances, sequencing technology also showed how miRNAs regulate the transcriptome on a genome-wide scale. One technique that aims to accomplish this is called crosslinking, ligation, and sequencing of hybrids (CLASH) [101]. CLASH builds conceptually on the cross-linking and immunoprecipitation (CLIP) technique that can be used to detect in vivo RNA-protein interactions by sequencing [101]. CLASH purifies tagged AGO proteins from cells and AGO-bound RNA-RNA duplexes are then trimmed by RNases. Interacting RNA strands are ligated together and the resulting chimeric molecules are ultimately sequenced [101]. This approach revealed a surprisingly high number of non-canonical miRNA-mRNA interactions that contained bulged or mismatched nucleotides [122].

More recently, advances in sequencing protocols enabled the investigation of the intracellular miRNA kinetics [123]. The consideration of the time variable (which is often neglected in RNA sequencing studies) enabled the authors to discover that miRNAs are among the fastest produced transcripts but at the same time belong to the longest-lived transcripts found in cells [123].

### 2.5. Scaffolding

Besides the above described roles of ncRNAs in the regulation of transcript production and translation, there is also a more indirect way in which ncRNAs can influence the transcriptome. lncRNAs, for example, have been shown to act as molecular scaffolds in cells [124]. The lncRNA HOTAIR binds proteins involved in chromatin modification for epigenetic silencing of genes [125]. It was previously known that HOTAIR was required for silencing of the HOXD locus in the genome [126]. Yet, only upon genome-wide chromatin IP of PRC2 and LSD1 in the presence (control) and absence (knockdown) of HOTAIR it became clear that HOTAIR was required to target epigenetic silencing proteins to hundreds of genes across the genome [125]. RNA in situ conformation sequencing (RIC-seq) provides another example of ncRNA scaffolding properties that were revealed by RNA sequencing [99]. This method, as well as the Mapping RNA interactome in vivo (MARIO) technology [100], are based on proximity ligation and allow for the investigation of intra- and intermolecular RNA–RNA interactions. The resulting three-dimensional interaction maps revealed that lncRNAs such as MALAT1 and NEAT1 extensively interact with highly expressed genes and thereby act as RNA hubs [99].

## 3. ncRNA in Human Health and Disease

As transcriptional and posttranscriptional regulators of gene expression, ncRNAs are involved in many cellular physiological and pathological processes. Thus, they represent a promising target in medical research. In particular, RNA sequencing has uncovered many ncRNAs that are differentially expressed in the context of various diseases. Accordingly, ncRNA research has the potential to elucidate pathological mechanisms, identify novel prognostic and diagnostic markers or represent therapeutic targets [127]. This potential for ncRNAs in these fields is particularly promising in the light of the fact that the majority of hits from genome-wide association studies (GWAS) mapped within non-coding regions [128]. For this purpose, it is essential to study ncRNAs and whole transcriptomes under healthy, physiological conditions. On the basis of this, pathological deviations can then be detected and provide information about disease development and progression.

### 3.1. Unraveling the ncRNA Landscape in Humans

In order to lay out the ncRNA landscape, sequencing approaches are needed to broadly uncover which ncRNAs are expressed in any given tissue. Thus, RNA sequencing greatly helped to unravel the transcriptomic landscape and thus generate reference atlases for comparison of pathological conditions (Figure 1). As an example, analysis of sequencing data discovered that circRNAs are highly prevalent in human cells, and that they are strongly induced during human fetal development [129,130]. Furthermore, completely new types of small ncRNAs have been discovered in multiple human cell types such as ncRNA-derived fragments, which exhibit regulatory functions independent of the parental transcripts [31,131,132] (Figure 1). Among these fragments, tRNA-derived fragments (tRFs) are of particular medical relevance, as they were shown to be involved in cell proliferation, apoptosis and protein translation [133]. Their differential expression in the context of various diseases including different kinds of cancer [133], ovarian endometriosis [134] and Alzheimer’s disease [135] suggests a role in disease development and progression.

In general, there are large differences in the abundance of ncRNAs within different cell types and tissues. Especially circRNAs and lncRNAs exhibit tissue-specific expression [136,137,138,139]. In order to obtain a complete picture of the transcriptomes, numerous projects are in progress to create databases and RNA atlases [47,64], thus creating collections of reference exomes can facilitate the search for differentially expressed ncRNAs in a pathological context (Table 2). One organ that has been poorly represented in the various RNA expression atlases is the placenta. Therefore, a recent study characterized the placenta transcriptome extensively in health as well as upon preeclampsia and fetal growth restriction [140]. Besides protein-coding transcripts, 679 circRNAs were present in 90% of the samples leading the authors to further investigate which circRNAs are functionally relevant. Interestingly, by using publicly available tandem mass spectrometry datasets the authors identified peptides that span the back-splice junction of the placental circRNAs, potentially suggesting that some placental circRNAs are not ncRNAs [140].

### 3.2. ncRNAs Associated with Pathological Mechanisms

Due to their complex regulatory functions, aberrantly expressed ncRNAs may contribute to the pathogenesis and progression of disease. This has been reported in the context of numerous pathologies in particular cancer [158] and neurodegenerative diseases [96,97]. Hence, the identification of ncRNAs is still an important computational challenge, in particular for non-linear RNAs such as circRNAs. Recently, deep learning based computational methods have been developed aiming at improving circRNA identification. Examples of such tools include Junction Encoder with Deep Interaction (JEDI) [159] or circDeep [160]. In addition, another tool, seek for differentially expressed CircRNAs in Transcriptome (seekCRIT) is dedicated to finding differentially expressed circRNAs [161]—making it a useful tool for the detection of differentially expressed genes when comparing diseased and healthy conditions.

Once identified, sequencing of circRNAs can also enable insights into their potential function based on their sequence. According to the competing endogenous RNA hypothesis [162], circRNAs as well as lncRNAs and pseudogene transcripts that share miRNA response elements with mRNAs can competitively bind these miRNAs. Consequently, they decrease the number of miRNAs available for targeting mRNAs. Such regulatory networks are increasingly discussed, for example, in the context of cancer initiation and various other malignant conditions [163,164,165]. For example, whole transcriptome sequencing identified that lncRNA LINC00473 is overexpressed in invasive pituitary adenoma. This lncRNA in turn binds miRNA miR-502-3p, resulting in the upregulation of KMT5A expression, which subsequently promotes proliferation and ultimately disease progression [165].

Interestingly, in addition to these endogenous interactions between e.g., miRNAs and their mRNA targets, there are also interactions between pathogen-derived RNAs and human host RNAs. In a recent study, Yang et al. report that the genomic RNA of the SARS-CoV2 virus interacts with a number of host RNAs [166]. Using sequencing of psoralen crosslinked, ligated, and selected hybrids (SPLASH) [102], the authors were able to identify more than 300 of these host RNAs. Beyond many mRNAs, they detected a high abundance of interactions with the small nucleolar RNA SNORD27. Interestingly, a sequencing approach that enabled mapping of 2′-O-methylation sites called Nm-seq [167] discovered a decrease in 2′-O-methylation in host RNA and an increase in viral RNA’s 2′O-methylation. Given that SNORD27 is responsible for 2′-O-methylation of 18s rRNA, the authors hypothesize that the interaction with SNORD27 is a mechanism to stabilize viral RNA and protect it from degradation via modifications.

### 3.3. Discovery of Diagnostic ncRNAs

Liquid biopsy is a promising approach in clinical medicine that involves the collection of body fluids for diagnostic and prognostic analysis and monitoring. The aim is to replace more invasive tissue sampling and examinations as well as to enable earlier diagnosis and continuous monitoring especially for diseases that benefit from early detection. In order to find biomarkers for these purposes, RNA, DNA or proteins can be isolated and analyzed from cells found in e.g., blood, bronchial lavage, urine or cerebrospinal fluid. Especially for the investigation of neurological diseases, liquid biopsies offer a promising opportunity, given that the human brain is one of the most difficult organs to access for tissue biopsy in the human body. These benefits are exemplified in a recent study which analyzed small RNA sequencing data obtained from Parkinson’s disease patients’ whole blood [168]. The authors discovered multiple candidate miRNAs as biomarkers, mostly originating from immune cells. These miRNAs were previously connected to systemic inflammation and mitochondrial dysfunction which are known hallmarks of Parkinson’s disease [169].

One advantage of analyzing RNA from whole blood samples is that it is technically rather simple, yet very informative. A recent study has demonstrated that whole blood transcriptome predicts gene expression for other tissues in the body [170]. Interestingly, for certain diseases such as type 2 diabetes, the whole blood transcriptome was almost as powerful as the actual measured tissue expression for predicting the disease state [170]. One downside, however, is that the large amount of blood cell derived RNA can potentially mask crucial information from the heavily diluted host tissue transcriptome. In these cases, one would want to enrich non-blood cell types, such as tumor cells circulating in the blood, which could then be analyzed even at single cell resolution [171]. Single cell sequencing of circulating tumor cells can also be informative for investigating the ncRNA profile of tumor cells in the blood as recently demonstrated for miRNAs [171].

In addition to these approaches, analysis of extracellular RNA emerges as a promising field for medical diagnostics. Sequencing cell-free RNA has demonstrated encouraging results for the diagnosis of several diseases ranging from cancer to cardiovascular diseases [172]. In contrast to protein-based biomarkers, a great advantage of RNA is that it can be amplified. In recent years technological developments enabled the amplification and sequencing of tiny amounts of RNA even from single cells [80]. Another advantage of RNA over DNA (which could also be amplified) is that it is continuously shed from cells. In contrast, DNA exits cells mostly when the cell is dying. Cell-free ncRNA in particular represents a promising diagnostic candidate pool, given that many ncRNAs are differentially expressed upon malignant transformation in human tissues [105,173]. Recently, Huang et al. performed RNA sequencing on serum-derived exosomes from hepatocellular carcinoma patients [174]. In comparison to healthy patients, thousands of differentially expressed mRNAs as well as lncRNAs were detected. Interestingly, lncRNAs showed higher expression levels, tissue specificity and lower expression variability compared to the differentially expressed mRNAs highlighting the diagnostic and prognostic potential of ncRNAs [174]. Along those lines, a pancreatic adenocarcinoma diagnostic signature consisting of five miRNAs was recently used to distinguish cancer patients from healthy individuals as well as from benign pancreatitis [175]. Interestingly, the miRNA signature was discovered by sequencing tumor tissue samples, yet the signatures could also be found in blood serum of patients [175]. While extracellular RNAs in blood are subject of many studies, comparatively few studies focus on other body fluids. To broaden the spectrum of biomarker searches, recently the Human Biofluid Atlas was introduced. This atlas contains sequencing data of mRNAs, small ncRNAs as well as circRNA from 20 different body fluids. This resource was built on an improved library preparation method for small ncRNAs [176]. The obtained data show large differences in the abundance of RNA biotypes between the body fluids, which underscores the importance of including ncRNAs in the search for diagnostic cell-free RNAs [176].

## 4. Exploring the ncRNA Landscape beyond Humans

Alongside advancements in the field of ncRNA research in human health and disease, in recent years there was also a surge in research of non-human ncRNAs and their potential roles. Thanks to new sequencing methods and novel approaches to data analysis, it has become possible to elucidate ncRNAs and their roles in viruses and various organisms ranging from archaea over scorpions to mice.

Here, we provide a general overview of some of the recent advancements in research fields beyond human biology with the help of sequencing technology and computational analysis.

### 4.1. Viruses

Viral ncRNA studies are a rapidly evolving research area that was propelled by the rise of sequencing technologies [177]. Since the viral genome is size constrained, any ncRNA present must carry out a vital task, such as evasion of host immunity. One example is human cytomegalovirus (HCMV) miR-UL112, which targets major histocompatibility complex class I-related chain B gene expression and thereby reduces the chance of the infected cell being killed by natural killer cells [178]. Additionally, as RNAs are less likely to elicit an immune response compared to proteins, in some cases ncRNA generation is more advantageous for viruses than protein synthesis [179]. One example of the impact of sequencing on viral ncRNA research is the development of a new method to study the interactions of an ncRNA expressed in herpesvirus. This method integrated psoralen-mediated RNA-RNA crosslinking with sequencing and resulted in the identification of viral ncRNA targets, highlighting its effect on inhibition of host cell apoptosis [180].

More recently, RNA-seq was used to assess the ncRNA repertoire produced by Ebola virus (EBOV) and Marburg virus. It was found that EBOV encodes small ncRNAs that act independently of the host miRNA machinery and do not display the ability to suppress viral replication or silence gene expression, thus leaving the question of their role in the host cell open [181].

Besides human viruses, other viral pathogens have also been investigated. A recent study examined the African Swine Fever virus (ASFV), which lethally infects pigs going along with substantial economic ramifications [182,183]. Several novel small ncRNAs of viral origin were discovered, one of which was shown to repress viral reproduction. Considering the absence of an effective vaccine against ASFV, this study added valuable information about the host-virus interactions, which may help in ASFV vaccine development [182].

### 4.2. Bacteria and Archaea

Sequencing has also opened up new horizons in prokaryotic ncRNA research. The development of metatranscriptomics has proven particularly useful, wherein the transcriptome of a complex microbiome is sequenced as a whole. This not only makes it possible to detect even lowly expressed genes, such as some ncRNAs but enables the investigation of non-culturable organisms using high-throughput sequencing [184,185]. The latter point is especially significant as only a small proportion of the microbial species are culturable at this point. Yet, the ability to study non-culturable microorganisms is important for both ecological (e.g., bioremediation) [185], and medical (e.g., human microbiota) research [186].

An important class of ncRNAs found in prokaryotes are small RNAs (sRNA) that range in length from 50–500 nt and regulate gene expression through RNA-RNA interactions [187]. They were described in bacteria even before the discovery of the first miRNAs in eukaryotes [188,189,190]. Yet, only recently with advances in high-throughput sequencing was it possible to study them more comprehensively. sRNAs can be broadly classified into trans- and cis-encoded sRNAs [187]. The former are also called intergenic sRNAs (itsRNAs) and are able to interact with several genes, mainly regulators of transcription [187], while the cis-encoded antisense RNAs (asRNAs) can target only the gene (e.g., transposons) that is transcribed on the DNA strand opposite to them [187,191].

A recent study looked at the metatranscriptomic landscape of extremophiles inhabiting halite nodules (salt rocks) in the Atacama desert. Most of the extremophiles belonged to the domain of Archaea and specifically the class of *Halobacteria*. Shotgun metagenomic sequencing was employed and resulted in the identification of over 1500 ncRNAs, 54% of which were of archaeal origin. Interestingly, significantly more intergenic sRNAs were found in archaea than in bacteria and the reverse was observed for antisense sRNAs [187].

### 4.3. Plants

The rise of sequencing technologies has also enabled more specific and focused plant-based studies of ncRNAs. A particular focus has been laid on the role of ncRNAs in plant immunity, development and stress response (e.g., abiotic factors) [192,193]. Sequencing has also facilitated the study of plants not commonly used in research, such as pistachio [194], sugar beet [195], and kiwifruit [196]. Even in a well studied plant such as Arabidopsis thaliana, sequencing has allowed to uncover a large number of previously undescribed lncRNAs and in particular highlighted the role of cold-induced lncRNA MAS in preventing precocious flowering [197].

Generally, studies of plants used as crops have great economic significance and are therefore of particular value to society. Notably, crop species such as Chinese cabbage and rice have come under scrutiny recently. By employing NGS based approaches (e.g., small RNA sequencing) the roles of ncRNAs in conferring resistance against infections [198,199,200], and in adapting to environmental factors (e.g., heat resistance) [201] have been highlighted.

A good example of a specialized sequencing approach being used in plant ncRNA research is the study by Ariel et al. where by employing CHiRp sequencing the function of lncRNA Auxin-Regulated Promoter Loop (APOLO) was studied in *Arabidopsis thaliana* [202]. The results indicated that APOLO acts on distant targets in trans not only through sequence complementarity but also by R-loop formation and conformational changes of chromatin [202].

Further advancements in ncRNA research in plants have been made by the use of long-read sequencing technology. One example is the study of lncRNA expression in seed development of wheat and triticale (hybrid of wheat and rye) by nanopore sequencing. The study highlighted the utility of nanopore sequencing for genomes with high complexity and identified a large number of novel lncRNAs [203]. In the field of plant genomics, long-read sequencing can help to avoid biases regularly seen in short-read RNA-seq studies [203].

Finally, considering the characteristics specific to plant lncRNAs [204,205,206] recent effort has been made to develop plant specific lncRNA prediction tools, examples of which are PlncRNA-HDeep [207] and LncMachine [208]. Plant circRNA discovery also poses challenges, as their sequences have unique features, such as introns flanking plant circRNA exons containing fewer repetitive and reverse complementary sequences than in the case of animal circRNAs [209]. These features need to be taken into account, which sparked the development of plant specific circRNA identification tools such as PCirc [210], or CircPlant [211].

### 4.4. Fungi

ncRNAs account for up to 25% of the genome of budding yeast (*Saccharomyces cerevisiae*), one of the most studied fungi species, with many ncRNAs acting as important gene expression regulators [212]. The development of sequencing technologies has had an important role for the fungi research field. For instance given the importance of antisense transcription of ncRNAs in fungi, specifically budding yeast, strand-specific RNA-seq has been integral for fungal ncRNA studies [213,214,215]. The study of five yeast species revealed that a number of antisense transcripts are highly conserved over 150 million years of evolution [215].

Recently, RNA sequencing has been used to assess the effect of ncRNA deletion on gene expression in yeast [212], as well as the discovery of 17 novel structural ncRNAs conserved amongst five fungal species [216]. Another highlight is the discovery of a positive lncRNA regulator of carotenoid production in fungi of the genus *Fusarium* [217]. This is noteworthy as some species of *Fusarium* are used as models to study carotenoid production [159], which is important for biotechnological applications of carotenoids as food colorants, animal feed, and nutraceuticals [218].

### 4.5. Protists

The advancements in sequencing technologies have also accelerated the studies of protists. The evolutionary distance between protists and the much more studied model eukaryotes makes the interpolation of findings from other species difficult for protist research [219], and hence, addressing research questions by directly studying protists is of great importance for the field.

A good example showcasing the importance of the increasing amount of sequencing data is a recent article examining the role of lncRNAs in protist parasites of the genus *Cryptosporidium*. This parasite is one of the main causes of diarrhea in young children and a noteworthy cause of mortality [220]. In the study using publicly available sequencing data on several *Cryptosporidium* species from different developmental stages, the authors were able to characterize the *Cryptosporidium* lncRNAs. It was found that nearly 10% of the annotated mRNAs have corresponding antisense lncRNA and lncRNA expression was positively correlated with the expression of upstream mRNAs. This might suggest the presence of bidirectional promoters or lncRNAs acting as positive regulators of mRNA expression. In total, almost 400 novel lncRNAs were identified with the vast majority being differentially expressed between developmental stages [220].

### 4.6. Invertebrates 

Invertebrates comprise ~95% of animal species [221], and almost 80% of the invertebrate genomes are considered to not code for proteins [222]. With the rise of sequencing technologies, it has become possible to start to examine this vast pool of species on a genomic level. The Global Invertebrate Genomic Alliance (GIGA) consortium for example aims to standardize and promote sequencing of invertebrate species [221]. In addition, the readily available RNA-seq datasets are propelling invertebrate studies, like for example a more in-depth look at the lncRNA transcriptome of the invasive species of codling moth [223], or the characterization of age-dependent expression changes of tRF in *Caenorhabditis elegans* [224]. RNA sequencing of developing *Drosophila* tissue identified the natural antisense transcript bsAS as one of the most highly expressed lncRNAs in the fly wing [225]. This ncRNA is responsible for inducing the expression of neural specific genes as identified by RNA sequencing upon bsAS knockout [225]. Similarly, a study of the mosquito *Aedes albopictus* (a carrier for Dengue and Zika viruses) took advantage of readily available sequencing data. By combining datasets with data generated by themselves, the authors were able to identify more than 10,000 novel lncRNAs in *A. albopictus* including ncRNAs with potentially modulating effects on Dengue and Zika virus replication [226]. Studies like these set the stage for a more thorough analysis of ncRNAs in invertebrate human parasites and vectors of dangerous diseases, potentially revealing therapeutic targets.

Another recent study investigated the poorly defined lncRNA landscape of scorpions, in particular, the venom glands of the species *Androctonus crassicauda* [227]. In the course of the study, authors developed a computational pipeline suitable for distinguishing protein-coding from non-protein-coding genes in arthropod species lacking a reference genome. The results of predicted scorpion lncRNAs showed that most did not share orthologs even with closely related species and were characterized by, e.g., lower GC content or shorter transcript length. In total, 12,642 potentially novel lncRNAs were described, highlighting the important role of ncRNAs in scorpion venom glands [227]. This study exemplifies that a surprisingly large number of ncRNAs and their function are yet to be discovered in invertebrates.

### 4.7. Vertebrates

In comparison to other taxa, the state of ncRNA research is more advanced in vertebrates. Mammalian organisms, in particular rodents and humans, have been studied extensively. But other vertebrates have traditionally not been the focus of vertebrate research. Examples include the study of the repertoire and function of circRNAs in the liver of the Whitespotted Bamboo shark [228], and the identification of miRNAs and lncRNAs important in the reproduction of Chinese soft-shelled turtle [229].

Amongst avians, chicken has been extensively researched. Studies utilizing sequencing technologies have focused on the roles ncRNAs play in embryogenesis [230], environmental adaptation [231], and tissue specific expression [232,233]. Chicken diseases have also received attention with the expression profiles of lncRNAs being studied in avian infectious bronchitis coronavirus (IBV) infected chicken macrophages [234]. The improved characterization of cellular consequences of IBV infection will increase our understanding of the disease, which is an important factor for the poultry industry [234].

Studies focused on livestock represent another example of understudied vertebrate research given its large economic impact. An example is the investigation of ncRNAs in cattle considering that cattle and buffalo meat and milk are the sources of 45% of global animal protein supply [235]. Computational analysis of sequencing data has already found lncRNAs associated with metabolism in cattle [236]. Furthermore, lncRNAs are responsible for differences in muscle characteristics between cattle and buffalo [237], which complements a previous study of skeletal-muscle related bovine lncRNAs [238]. It has also been suggested that some circRNAs by acting as miRNA sponges potentially influence insulin-like growth factor 1 receptor expression and subsequently muscle growth in cattle [239].

Beyond tackling food supply issues for a growing world population, the generation of transgenic animals for production of biopharmaceuticals presents another incentive for increased research efforts in vertebrates. One of the main methods used to this end is somatic nuclear cell transfer (SCNT) [240], which still faces the issues of low efficiency [241]. To address this challenge, a recent paper studied goat 8-cell SCNT embryos. The authors found the lncRNA lnc_3712 is crucial for the reprogramming efficiency by repressing the expression of the demethylase Kdm5b. The knockdown of lnc_3712 led to the increased expression of several vital embryonic genes and resulted in better development of goat SCNT embryos [241].

Interestingly, the increasingly broad application of sequencing to discover the ncRNA landscape of traditionally understudied species is not limited to species that are alive today. Ancient DNA sequencing offers insights into genomic landscapes of extinct life forms [242]. A recent study speculated about the role of lncRNAs in the emergence of creativity in *Homo sapiens.* By analyzing DNA sequencing data from *Homo sapiens*, Neanderthals and chimpanzees, the authors found that chimpanzees had no lncRNAs amongst the genes that have an association with modern human personality and a much higher proportion of lncRNAs was detected in *Homo sapiens* compared to Neanderthals. In total, 94% of those genes found only in *Homo sapiens* were ncRNAs, most being lncRNAs [243].

Although most of the above described findings were based on short-read sequencing technology, the development of long-read sequencing has also left its mark on mammalian research. For instance by employing nanopore sequencing, human and mouse brain circRNAs were characterized, revealing widespread splicing events in circRNAs, highlighting the importance of developing sequencing technologies [244]. It will be interesting to see how long-read sequencing technology will benefit the ncRNA research in other less well studied vertebrates in the future.

## 5. Concluding Remarks

The rise of sequencing technologies has revolutionized the study of the transcriptome, including ncRNAs. Much has been understood about ncRNAs and recent years have seen a rapid increase of published ncRNA papers (Figure 1). Thanks in part to the decreasing cost [245], sequencing technology has become an increasingly popular tool for the field of ncRNA research.

The development of single-cell sequencing has allowed the study of ncRNAs at the single-cell level and with the more specialized and targeted sequencing methods it has become possible to study not only the landscape of ncRNAs but also their functionalities in the cell. The recently developed long-read sequencing technology demonstrated its potential for the field of ncRNA research, for example by elucidating circRNA exon composition [244]. These technical advances will not only benefit the discovery of medically relevant ncRNAs as well but also enrich our basic biological understanding of diverse species. Sequencing has also resulted in the accumulation of an ever-growing amount of information. Hence, the creation of various databases focused on specific ncRNA species, functions or organisms will enable a comprehensive analysis of the ncRNA landscape within and across species boundaries (Table 1). Similarly, computational tools are getting more sophisticated and recently, deep learning-based approaches have enriched the available repertoire of tools for analyzing ncRNA sequencing data.

Although the field of ncRNA sequencing has come a long way since the discovery of the first ncRNAs in the 1950s and 1960s, exciting technological advances will continually advance our understanding of the complexity of the ncRNA transcriptome. For instance, the performance of nanopore sequencing technology has been steadily improving in the last years [246]. The ability of this technology to detect various modified nucleotides could help us to uncover the true molecular variability of the pool of ncRNA in various cell types and organisms. It is conceivable that taking the modification status of ncRNAs into account might increase their prediction accuracy for medical diagnostics and disease outcome.

Nevertheless, challenges remain. With the discovery of novel ncRNA species, increased efforts will be needed to elucidate their functions *in vivo*. Detection of ncRNAs still remains challenging. Reasons for this include the low expression levels of many ncRNAs [79], unique features of certain ncRNAs (e.g., circRNA, tRNAs) and the bias RNA-seq library preparation methods and computational pipelines can introduce. For example, reverse transcriptases can generate spurious circular RNA molecules confounding the in silico analysis of circRNAs [247]. Due to the distinct secondary structures and nucleoside modifications, tRNA sequencing is still a major challenge and requires further development of library preparation protocols and analysis workflows as described by Behrens et al. [248], Thomas et al. [249] and Warren et al. [250]. Similarly, depleting rRNA and excluding reads mapping to unwanted RNAs may result in missing a number of ncRNA species such as rRNA and tRNA derived fragments (rRFs and tRFs) [251,252]. Annotation of ncRNAs in sequencing data still lacks uniformity and accuracy [253]. The latter can lead to erroneous identification of ncRNA species, which in particular is the case for piRNAs [254,255]. Furthermore, the plethora of available computational tools need to be properly benchmarked and the results of in silico analysis validated experimentally. Many species still lack comprehensive reference genomes impeding the study of their ncRNA transcriptome. Above all, methods for validating the functional relevance of the newly discovered ncRNAs are very sought after, given that it remains a challenge to distinguish them from transcriptional noise. Coming years will likely see continuous efforts to address these challenges in order to enlighten our understanding of what we used to call the genomic dark matter.

## Figures and Tables

**Table 1:** A collection of recently developed or updated non-coding RNA databases containing NGS generated data. All URLs were accessed on 22 October 2021.

Name	Species	Database Content	URL	Refs.
AlnC	682 Angiosperms	lncRNA	http://www.nipgr.ac.in/AlnC	[34]
ASRA	21 Vertebrates	Circulating sncRNA	https://ccb-web.cs.uni-saarland.de/asra/	[35]
circ2Go	Human	circRNA	https://circ2go.dkfz.de/	[36]
CircAtlas 2.0	6 Vertebrates	circRNA	http://circatlas.biols.ac.cn/	[37]
circBank	Human	circRNA	http://www.circbank.cn/	[38]
circBase	3 Vertebrates 2 Invertebrates	circRNA	http://www.circbase.org/	[39]
circFunBase	7 Plants 8 Vertebrates 1 Invertebrate	circRNA	http://bis.zju.edu.cn/CircFunBase/index.php	[40]
CIRCpedia 2.0	4 Vertebrates 2 Invertebrates	circRNA	https://www.picb.ac.cn/rnomics/circpedia/	[41]
DeepBase v3.0	11 Vertebrates 2 Invertebrates	ncRNA	https://rna.sysu.edu.cn/deepbase3/index.html	[42]
DIANA-lncBase v.3	2 Vertebrates	miRNA targets on non-coding transcripts	https://diana.e-ce.uth.gr/lncbasev3/home	[43]
DIANA-Tarbase v8	2 Viruses 7 Plants 2 Invertebrates 7 Vertebrates	miRNA-gene interactions	http://www.microrna.gr/tarbase	[44]
EVAtlas	Human	Extracellular vesicle ncRNA	http://bioinfo.life.hust.edu.cn/EVAtlas/	[45]
exoRBase 2.0	Human	Exosome ncRNA	http://www.exorbase.org/	[46]
GTEx version 8	Human (tissue)	Transcriptome	https://gtexportal.org/home/	[47]
Lantern	Human	lncRNA	https://sysbio.lab.iupui.edu/lantern/	[48]
LncATLAS	Human	lncRNA subcellular localization	https://lncatlas.crg.eu/	[49]
LncBook	Human	lncRNA	https://ngdc.cncb.ac.cn/lncbook/index	[50]
LncExpDB	Human	lncRNA	https://ngdc.cncb.ac.cn/lncexpdb/	[51]
LNCipedia 5	Human	lncRNA	https://lncipedia.org/	[52]
lncRNAKB	Human	lncRNA	http://psychiatry.som.jhmi.edu/lncrnakb/	[53]
LncSEA	Human	lncRNA	http://bio.liclab.net/LncSEA/index.php	[54]
MINTbase 2.0	Human	tRF	https://cm.jefferson.edu/MINTbase/	[55]
miRBase	271 organisms	miRNA	https://www.mirbase.org/	[56]
MiREDiBase	4 Vertebrates	Editing events in miRNA	https://ncrnaome.osumc.edu/miredibase/	[57]
MirGeneDB 2.0	22 Vertebrates 23 Invertebrates	miRNA	https://mirgenedb.org/	[58]
mirTarBase 9.0	16 Vertebrates 3 Invertebrates 5 Plants 3 Viruses	miRNA-target interactions	https://mirtarbase.cuhk.edu.cn/~miRTarBase/miRTarBase_2022/php/index.php	[59]
miRWalk	6 Vertebrates	miRNA binding sites	http://mirwalk.umm.uni-heidelberg.de/	[60]
NONCODE v6.0	23 Plants 1 Fungus 2 Invertebrates 13 Vertebrates	ncRNA	http://www.noncode.org/index.php	[61]
riboCIRC	2 Invertebrates 4 Vertebrates	Translatable circRNA	http://www.ribocirc.com/	[62]
Rfam 14.6	Various	RNA families	https://rfam.org/	[63]
RNA Atlas	Human (tissue and cell lines)	Transcriptome	http://r2platform.com/rna_atlas	[64]
piRBase 3.0	28 Invertebrates 16 Vertebrates	piRNA	http://bigdata.ibp.ac.cn/piRBase/	[65]
piRNAclusterDB 2.0	23 Invertebrates 28 Vertebrates	piRNA clusters	https://www.smallrnagroup.uni-mainz.de/piRNAclusterDB/	[66]
piRTarBase	2 Invertebrates	piRNA targeting sites	http://cosbi6.ee.ncku.edu.tw/piRTarBase/	[67]
PlantcircBase 6.0	20 Plants	circRNA	http://ibi.zju.edu.cn/plantcircbase/index.php	[68]
PtRFdb	10 Plants	tRF	http://14.139.61.8/PtRFdb/index.php	[69]
snoDB	Human	snoRNA	http://scottgroup.med.usherbrooke.ca/snoDB/	[70]
TarDB	43 Plants	miRNA targets	http://www.biosequencing.cn/TarDB/	[71]
TransCirc	Human	Translatable circRNA	https://www.biosino.org/transcirc/	[72]
TSCD	2 Vertebrates	circRNA	http://gb.whu.edu.cn/TSCD/	[73]

**Table 2:** A collection of recently developed or updated medically relevant non-coding RNA databases containing NGS generated data. All URLs were accessed on 22 October 2021.

Name	Disease/Pathogens	Database Content	URL	Refs.
CircRic	Cancer	circRNA	https://hanlab.uth.edu/cRic/	[141]
CircRNADisease	Various	circRNA	http://cgga.org.cn:9091/circRNADisease/	[142]
CSCD2	Cancer	circRNA miRNA	http://geneyun.net/CSCD2/	[143]
dbDEMC 3.0	Cancer	miRNA	https://www.biosino.org/dbDEMC/index	[144]
DeepBase v3.0	Cancer	ncRNA	https://rna.sysu.edu.cn/deepbase3/index.html	[42]
Lnc2Cancer 3.0	Cancer	lncRNA circRNA	http://www.bio-bigdata.net/lnc2cancer/	[145]
LncRNADisease 2.0	Various	lncRNA circRNA	http://www.rnanut.net/lncrnadisease/	[146]
LncRNASNP2	Various	lncRNA	http://bioinfo.life.hust.edu.cn/lncRNASNP2	[147]
LncTarD	Various	lncRNA miRNA	http://bio-bigdata.hrbmu.edu.cn/LncTarD/index.jsp	[148]
MiOncoCirc	Cancer	circRNA	https://mioncocirc.github.io/	[149]
mnDR v3.1	Various	ncRNA	http://www.rna-society.org/mndr/	[150]
ncRNA-eQTL	Cancer	Single nucleotide polymorphism (SNP) effects on ncRNA	http://ibi.hzau.edu.cn/ncRNA-eQTL/	[151]
ncRPheno	Various	ncRNA	http://www.liwzlab.cn/ncrpheno/ncrpheno.html	[152]
NSDNA	Nervous system disorders	ncRNA	http://bio-bigdata.hrbmu.edu.cn/nsdna/	[153]
PATHOgenex	Bacteria	Transcriptome	http://www.pathogenex.org/	[154]
TANRIC 2.0	Cancer	lncRNA	https://ibl.mdanderson.org/tanric/_design/basic/main.html	[155]
TCGA	Cancer	Transcriptome	https://portal.gdc.cancer.gov/	-
ViRBase v3.0	Viruses	ncRNA	http://www.rna-society.org/virbase/	[156]
VirusCircBase	Viruses	circRNA	http://www.computationalbiology.cn/ViruscircBase/home.html	[157]

## Data Availability

Not applicable.
